# Prevalence and Geographic Patterns of Self-Reported Short Sleep Duration Among US Adults, 2020

**DOI:** 10.5888/pcd20.220400

**Published:** 2023-06-29

**Authors:** Magdalena M. Pankowska, Hua Lu, Anne G. Wheaton, Yong Liu, Benjamin Lee, Kurt J. Greenlund, Susan A. Carlson

**Affiliations:** 1 Oak Ridge Institute for Science and Education, Division of Population Health, Centers for Disease Control and Prevention, Atlanta, Georgia; 2 Division of Population Health, National Center for Chronic Disease Prevention and Health Promotion, Centers for Disease Control and Prevention, Atlanta, Georgia

## Abstract

We estimated the prevalence of short sleep duration (<7 hours per day) among US adults aged 18 years or older by using 2020 Behavioral Risk Factor Surveillance System data. Nationally, 33.2% of adults reported short sleep duration. We identified disparities across sociodemographic characteristics, including age, sex, race and ethnicity, marital status, education, income, and urbanicity. Counties with the highest model-based estimates of short sleep duration clustered in the Southeast and along the Appalachian Mountains. These findings identified subgroups and geographic areas in which tailored strategies for promotion of optimal sleep duration (≥7 hours per night) are most needed.

**Summary**

**What is already known on this topic?**

Short sleep duration (<7 hours for adults) is associated with an increased risk of chronic conditions, yet one-third of US adults report short sleep duration.

**What is added by this report?**

Disparities in the prevalence of short sleep duration were identified across age, sex, race and ethnicity, marital status, education, income, and urbanicity. Counties with the highest model-based estimates clustered in the Southeast and along the Appalachian Mountains.

**What are the implications for public health practice?**

Findings highlight subgroups and geographic areas in which disparities in short sleep duration exist. Combining model-based local estimates of short sleep duration with neighborhood-level data and context can inform the development and implementation of tailored efforts to promote sleep health.

## Objective

Short sleep duration (sleeping <7 hours per 24-hour period) is associated with an increased risk of chronic conditions (eg, obesity, diabetes, hypertension, heart disease, stroke, anxiety, depression) (1). Increasing the proportion of adults who get enough sleep is a Healthy People 2030 objective (2). Yet in 2014, one-third of US adults reported short sleep duration (3). The prevalence of short sleep duration can vary by state, with a higher prevalence clustered in the southeastern US (3); however, less is known about trends by urbanicity and the clustering of short sleep duration at the county level. We examined the prevalence of short sleep duration among adults aged 18 years or older nationally by sociodemographic characteristics (ie, age, sex, race and ethnicity, marital status, education, and annual household income) and geographic characteristic (urban–rural classification) and identified geographic patterns of short sleep duration at the county level.

## Methods

We analyzed data from the 2020 Behavioral Risk Factor Surveillance System (BRFSS) to estimate crude and age-adjusted (4) short sleep duration prevalence nationally (50 states and the District of Columbia) and by age group, sex, race and ethnicity, marital status, education, annual household income, and urban–rural classification. BRFSS is an annual, state-based, random-digit–dialed landline and cell phone survey used to monitor health conditions and behaviors of noninstitutionalized adults aged 18 years or older in all 50 states, the District of Columbia, and participating US territories (5). The median response rate for the 50 states and the District of Columbia was 47.6% (range, 34.5%–67.2%) in 2020. We considered responses of less than 7 hours to the question “On average, how many hours of sleep do you get in a 24-hour period?” as reporting short sleep duration. We included data from respondents surveyed in all 50 states and the District of Columbia; information on sleep was reported by 390,193 (98.8%) respondents. We obtained Federal Information Processing Series codes for county of residence through a data-use agreement with BRFSS. Counties were classified into 6-level urban–rural classifications by using the National Center for Health Statistics 2013 classification scheme (6). We used trend tests to determine associations between the prevalence of short sleep duration and annual household income and urban–rural classification. We used pairwise *t* tests to compare the prevalence between subgroups across other characteristics. All comparisons reported were significant at *P* < .05.

We estimated the county-level crude and age-adjusted (4) prevalence of short sleep duration in 3,143 counties by using multilevel logistic regression and poststratification (MRP) and the Centers for Disease Control and Prevention’s PLACES approach (7,8). We constructed a multilevel regression model using 2020 BRFSS individual-level data on sex, age, race and ethnicity, and education level, and county-level data on those living below 150% of the poverty threshold from the 5-year 2016–2020 American Community Survey as well as state- and nested county-level random effects (8). We then applied predicted probabilities to county populations by using Census Vintage 2020 population estimates to generate the final predicted county-level prevalence estimates of short sleep duration. Estimates were validated by comparing crude model-based estimates with weighted direct survey estimates from counties with a sample size of 500 or more (n = 183) in BRFSS; the Pearson correlation coefficient was 0.90. We visualized the distribution of county-level prevalence estimates by quintiles. We used SAS version 9.4 (SAS Institute Inc) and SAS-callable SUDAAN version 11.0.3 (RTI International) to conduct all analyses. We used Esri ArcMap version 10.8.1 to create maps.

## Results

Overall, an age-adjusted 33.2% of adults reported short sleep duration in 2020 (Table). By age, adults aged 25 to 44 years; by sex, men; by education, those with some college; and by marital status, those who were divorced, widowed, or separated had the highest prevalence of short sleep duration. Non-Hispanic Native Hawaiian or Pacific Islander and non-Hispanic Black adults had a higher prevalence of short sleep duration compared with non-Hispanic White, non-Hispanic Asian, and Hispanic adults. The prevalence of short sleep duration increased with decreasing annual household income, from 29.3% (≥$75,000) to 38.1% (<$15,000), and decreasing urbanicity, from 32.0% (large central metropolitan counties) to 35.0% (noncore counties).

**Table:** Crude and Age-Adjusted Prevalence of Short Sleep Duration Among Adults Aged ≥18 Years, by Sociodemographic and Geographic Characteristics, Behavioral Risk Factor Surveillance System, US, 2020^a^

Characteristic	Unweighted no. of respondents	Crude prevalence, % (95% CI)	Age-adjusted prevalence, % (95% CI)
**Overall**	390,193	32.7 (32.4–33.1)	33.2 (32.8–33.6)
**Age group, y^b^**			
18–24	24,891	29.8 (28.7–31.0)	NA
25–44	93,327	36.4 (35.8–37.1)	NA
45–64	136,052	34.5 (33.9–35.1)	NA
≥65	135,923	26.0 (25.4–26.6)	NA
**Sex^c^**			
Female	211,071	32.1 (31.6–32.6)	32.6 (32.0–33.1)
Male	179,122	33.3 (32.8–33.9)	33.8 (33.3–34.3)
**Race and ethnicity^d^**			
Hispanic	30,885	32.1 (30.9–33.3)	32.0 (30.8–33.3)
Non-Hispanic American Indian or Alaska Native	6,787	38.5 (35.6–41.4)	38.5 (35.7–41.4)
Non-Hispanic Asian	9,396	30.5 (28.3–32.8)	30.8 (28.5–33.3)
Non-Hispanic Black	29,597	43.5 (42.4–44.6)	43.6 (42.4–44.7)
Non-Hispanic Native Hawaiian or Pacific Islander	1,246	46.5 (41.2–52.0)	46.5 (41.0–52.1)
Non-Hispanic White	294,308	30.7 (30.3–31.0)	31.8 (31.4–32.2)
Non-Hispanic multiracial	8,054	39.5 (37.2–41.9)	39.8 (37.5–42.3)
Non-Hispanic other	3,488	36.8 (33.5–40.2)	36.6 (33.2–40.1)
**Marital status^e^**			
Married or member of an unmarried couple	217,202	30.3 (29.8–30.8)	31.0 (30.4–31.5)
Divorced, widowed, or separated	99,926	37.4 (36.7–38.2)	41.6 (40.0–43.2)
Never married	69,484	34.5 (33.7–35.3)	36.4 (35.5–37.3)
**Education^f^**			
Less than high school diploma	24,634	33.7 (32.4–35.0)	33.7 (32.4–35.1)
High school graduate	103,526	34.6 (34.0–35.3)	35.8 (35.1–36.5)
Some college	108,508	35.8 (35.2–36.5)	36.8 (36.1–37.6)
College graduate or higher	151,840	27.2 (26.7–27.8)	27.0 (26.5–27.6)
**Annual household income, $^g^**			
<15,000	24,361	38.0 (36.4–39.5)	38.1 (36.5–39.7)
15,000 to <25,000	46,410	37.1 (36.1–38.2)	37.9 (36.8–39.0)
25,000 to <35,000	30,426	35.2 (33.8–36.5)	36.3 (34.9–37.7)
35,000 to <50,000	42,969	35.1 (34.0–36.2)	36.3 (35.2–37.5)
50,000 to <75,000	51,738	33.7 (32.6–34.7)	34.1 (33.1–35.2)
≥75,000	117,658	29.7 (29.1–30.3)	29.3 (28.6–29.9)
Missing	76,631	30.2 (29.5–30.9)	31.3 (30.5–32.1)
**Urban-rural classification^h^**			
Large central metropolitan	58,174	32.0 (31.2–32.8)	32.0 (31.2–32.9)
Large fringe metropolitan	76,295	32.7 (32.0–33.3)	33.2 (32.5–33.9)
Medium metropolitan	80,761	33.0 (32.3–33.6)	33.7 (33.0–34.4)
Small metropolitan	54,457	33.4 (32.5–34.3)	34.5 (33.6–35.5)
Micropolitan	61,818	33.6 (32.9–34.4)	34.8 (34.0–35.7)
Noncore	58,688	33.6 (32.6–34.5)	35.0 (33.9–36.0)

Abbreviation: NA, not applicable.

a Crude and age-adjusted prevalence and 95% CIs were directly estimated by using sampling weights. Includes data from the 50 US states and the District of Columbia. Age-adjusted estimates were standardized to the 2000 projected US population aged ≥18 years in 4 groups (18–24, 25–44, 45–64, ≥65) for all characteristics except age group (https://www.cdc.gov/nchs/data/statnt/statnt20.pdf). Categories may not sum to sample total because of missing responses.

b Significant difference in crude prevalence across all age-group comparisons assessed by pairwise *t* tests; *P* <.05 considered significant.

c Significant difference in crude and age-adjusted prevalence between male and female assessed by pairwise *t* tests; *P* <.05 considered significant.

d Significant differences in crude and age-adjusted prevalence found for most pairwise comparisons across racial and ethnic subgroups (assessed by pairwise *t* tests and *P* <.05 considered significant). Pairwise differences were not significant for the comparison of crude and age-adjusted prevalence for non-Hispanic White compared with non-Hispanic Asian; non-Hispanic Black compared with non-Hispanic Native Hawaiian or Pacific Islander; Hispanic compared with non-Hispanic Asian; non-Hispanic multiracial compared with American Indian or Alaska Native and non-Hispanic Other; and non-Hispanic American Indian or Alaska Native compared with non-Hispanic Other. Comparison of the age-adjusted prevalence was not significant, while the comparison of the crude prevalence was significant for non-Hispanic White compared with Hispanic.

e Significant difference in crude and age-adjusted prevalence across all marital status subgroup comparisons assessed by pairwise *t* tests (*P* <.05 considered significant).

f Significant differences in crude and age-adjusted prevalence found for most pairwise comparisons across education subgroups (assessed by pairwise *t* tests, *P* <.05 considered significant). Pairwise differences were not significant for the comparison of crude prevalence for “less than high school diploma” with “high school graduate.”

g Significant linear and quadratic trend in crude and age-adjusted prevalence using orthogonal polynomial contrasts trend tests (*P* <.05 considered significant). Indicates a nonlinear variation in addition to an overall increase as income attainment decreases.

h Urban–rural classification defined by the National Center for Health Statistics 2013 urban–rural classification scheme (www.cdc.gov/nchs/data_access/urban_rural.htm). Significant linear trend in crude and age-adjusted prevalence using orthogonal polynomial contrasts trend tests (*P* <.05).

Model-based age-adjusted county-level estimates of short sleep duration prevalence ranged from 23.8% (crude, 23.2%) in Boulder County, Colorado, to 48.4% (crude, 46.4%) in Greene County, Alabama. Overall, counties with crude and age-adjusted prevalence in the highest quintile were clustered in the Southeast and along the Appalachian Mountains (Figure).

**Figure:**
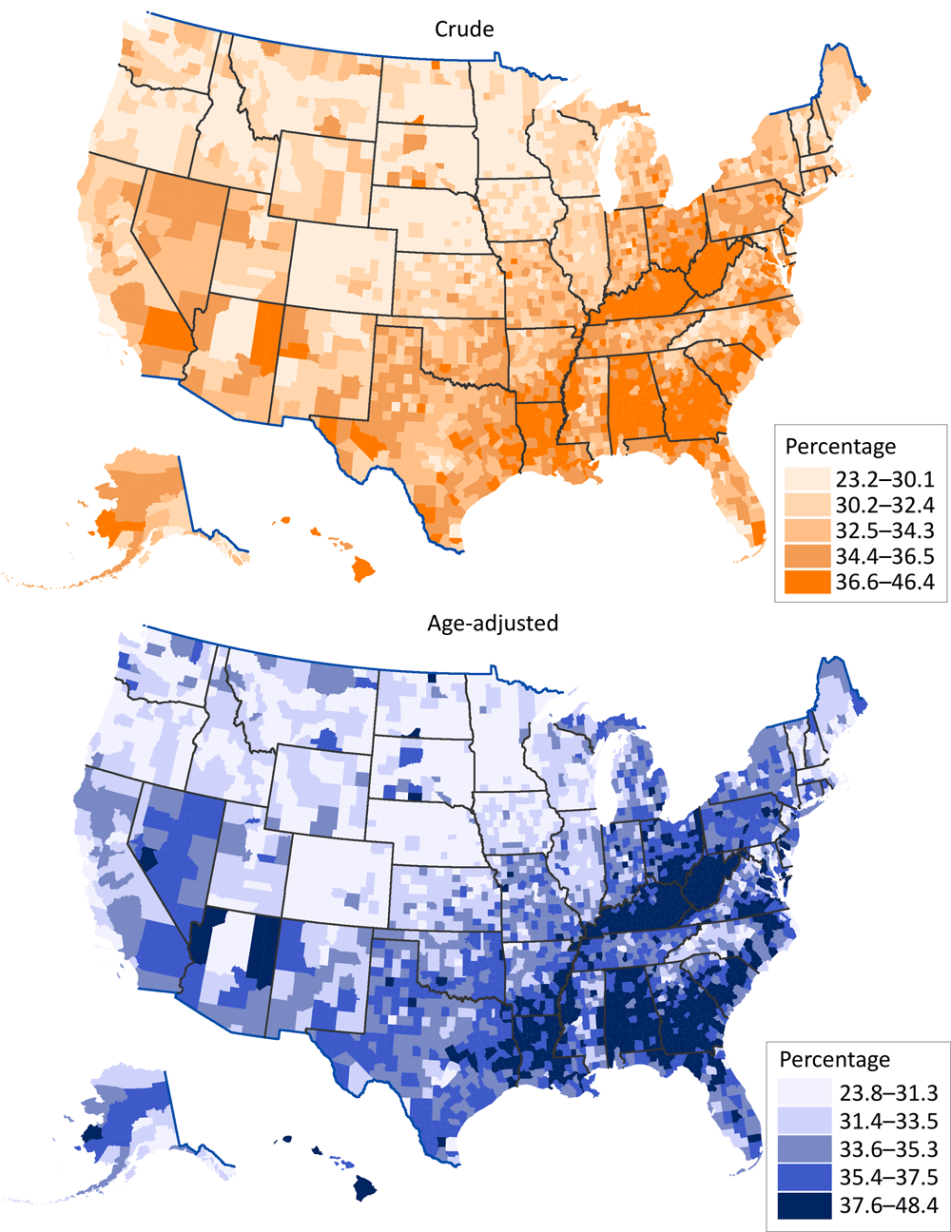
Model-based crude and age-adjusted county-level prevalence estimates of short sleep duration (<7 hours per 24-hour period) among adults aged 18 years or older, by quintile, United States, 2020. Urban–rural classification was defined by the National Center for Health Statistics 2013 urban–rural classification scheme (6). Age-adjusted estimates were standardized to the 2000 projected US population aged 18 years or older in 13 groups (18–24, 25–29, 30–34, 35–39, 40–44, 45–49, 50–54, 55–59, 60–64, 65–69, 70–74, 75–79, ≥80) (4). Data source: Centers for Disease Control and Prevention (7).

## Discussion

In 2020, one-third of US adults reported short sleep duration. Differences identified across sociodemographic characteristics, including age, race and ethnicity, education, and marital status, were similar to those identified in a previous study (3) and highlight the continued need for tailored strategies to address these disparities. By geographic characteristic, prevalence increased with decreasing urbanicity. In contrast to our study, a study using BRFSS data did not find urban–rural differences in the prevalence of sufficient sleep (9), but that study used data from 2013 and a definition of sleep duration that was different from ours. Similar to our results on sleep duration across the urban–rural continuum, previous studies found that the prevalence of health risk behaviors such as cigarette smoking and not meeting physical activity guidelines increased with decreasing urbanicity (9,10). Rural health may benefit from efforts that promote multiple health behaviors. For example, promoting regular physical activity can help establish healthy sleep habits and improve sleep duration (11).

Counties with the highest model-based prevalence of short sleep duration were clustered in the Southeast and along the Appalachian Mountains. The county-level geographic pattern of short sleep duration is similar to patterns of model-based estimates for the prevalence of diabetes, hypertension, heart disease, stroke, and depression, as well as mortality from heart disease and stroke (7,12). This similarity suggests that the geographic differences in short sleep duration may partially reflect geographic patterns of other chronic conditions, for which short sleep duration is a risk factor (1). Model-based estimates at the county level have been shown to be reliable (8) and are a valuable planning tool, especially when direct local data are unavailable. Our estimates offer a starting point for identifying and understanding geographic disparities, but additional neighborhood-level data and context can be incorporated into developing local efforts to promote sleep health. For example, examining and understanding the role of household and neighborhood factors (eg, sleeping conditions, safety, noise, light exposure) on sleep health (13) can help guide local public health practitioners in developing and implementing effective and tailored prevention activities, programs, and policies.

This study has several limitations. First, direct estimates were based on self-reported data and depended on accurate recall. Second, our results may have been influenced by nonresponse bias; we reduced this bias through the application of sampling weights. Third, the COVID-19 pandemic may have affected 2020 BRFSS data collection and potentially influenced estimates (5). Lastly, county-level estimates of short sleep duration were estimated by using MRP, which could introduce bias from the surveys (eg, recall, sampling) and modeling approach. Detailed limitations and strengths of MRP are addressed elsewhere (8).

Our findings suggest that promotion of sufficient sleep duration is needed in subgroups and geographic areas with a higher prevalence of short sleep duration. Combining model-based local estimates of short sleep duration with neighborhood-level data and context can further inform the development and implementation of tailored efforts to promote sleep health.
